# Assessment and Management of Eating Disorders at Community CAMHS in South Lanarkshire: A Quality Improvement Project

**DOI:** 10.1192/bjo.2024.414

**Published:** 2024-08-01

**Authors:** Tze Hui (Fifi) Phang, Sophie Hall, Amy Woodrow, Joseph Jameson, Raghuram Mahalingam Krishnasamy

**Affiliations:** 1 NHS Greater Glasgow and Clyde, Glasgow, United Kingdom; 2 NHS Lanarkshire, Hamilton, United Kingdom

## Abstract

**Aims:**

An evaluation of the service and care provided to eating disordered patients referred to Tier 3 CAMHS within NHS Lanarkshire. Eating disorders are recognised as a relatively common disease with preventable mortality. The primary aim was to determine if patients with eating disorders adhere to the assessment and management as outlined in MEED and SIGN 164. The secondary aim was to scope the number of eating disordered cases to plan recruitment and training of specialist staff.

**Methods:**

The pilot study was carried out in November 2022 and repeated in January 2024. The Electronic Patient Record and paper notes of eating disordered cases assessed in 2023 were used to audit against MEED and SIGN 164. Additional patient demographics including patient's age, sex, median BMI at initial appointment, working diagnosis and suspected co-morbidity were also collected. The service was further evaluated on its processes from source of referral, time taken to be seen, therapies offered and duration within service.

**Results:**

A total of 46 cases were identified in the audit compared to 57 in the pilot study. Most of the cases seen in 2023 were girls in their early teens (89% between the ages 13–16). 10% have a median % BMI <80%. 15 were given a diagnosis of AN (33%), 4 with BN (9%), 4 with ARFID (9%), 2 with OSFED (4%) and 19 with no formal diagnosis (42%). There was a high level of suspected comorbidity (80%).

Referrals were mostly made by GPs (87%), followed by school (11%) and other professionals (2%). The average time taken for the initial assessment was 63 days (40% were seen within 4 weeks). 14 (30%) of cases were offered FBT only whereas 3 (7%) had CBT-E. 7 (15%) did not receive any intervention and 19 (41%) were given other therapies.

With respect to the MEED risk markers, there had been improved recording of weight changes (40% to 80%), hydration status (40% to 70%), temperature (5% to 30%), bloods, over exercising (85% to 90%), purging (75% to 85%) and self-harm behaviours (85% to 90%). However there had been reduction in the recording of BP/HR (80% to 50%), ECG (75% to 40%) and engagement with services (75% to 60%).

**Conclusion:**

Overall, there's some improvement in assessment and management of ED cases but the standard remains inadequate. This project has helped understand the gaps in services and provisions available. Ongoing evaluation is required to help steer service development and optimise patient care.

